# Mental health, firearm ownership, and risk of death by suicide: a population-wide data linkage study.

**DOI:** 10.23889/ijpds.v7i3.1935

**Published:** 2022-08-25

**Authors:** Emma Ross, Dermot O'Reilly, Maguire Aideen

**Affiliations:** 1 Manitoba Centre for Health Policy

## Objectives

There is clear evidence from the USA that access to firearms increases suicide risk, but little equivalent evidence exists in the UK. The aim of the current study is to examine the risk of suicide and all-cause mortality for people in Northern Ireland (NI) who hold a licenced firearm.

## Approach

We link information on all registrations from the Firearms Certificate (FAC) Register between 2010-2020 to the health service population spine for NI residents born before 1st January 2005. Further linkage includes prescription medication data and death records with follow-up until 31st December 2020.

## Results

68,831 individuals held a FAC during the study period. FAC holders were more likely to be older, to reside in rural areas (OR 4.99, 4.89-7.83), and to come from more affluent areas (ORmost deprived 0.46, 0.43-0.50). During follow-up, 3,704 FAC holders died. 36 deaths were due to suicide, of which 16 were suicides by firearm. Only 23% of those who died by firearm suicide in NI were FAC holders. Preliminary findings indicate that after adjustment for age, area-level deprivation, and urbanicity, FAC holders had a lower risk of all-cause mortality (HR 0.64, 0.61-0.66) and death by suicide (HR 0.54, 0.39-0.76).

## Conclusion

In contrast to findings from previous studies, individuals with a licensed firearm were less likely to die by suicide.

## Acknowledgement

The authors would like to acknowledge the help provided by the staff of the Honest Broker Service (HBS) within the Business Services Organisation Northern Ireland (BSO). The HBS is funded by the BSO and the Department of Health (DoH). The authors alone are responsible for the interpretation of the data and any views or opinions presented are solely those of the author and do not necessarily represent those of the BSO.

